# The Clash of Two Epidemics: the Relationship Between Opioids and Glucose Metabolism

**DOI:** 10.1007/s11892-022-01473-0

**Published:** 2022-05-20

**Authors:** Laura L. Koekkoek, Luna L. van der Gun, Mireille J. Serlie, Susanne E. la Fleur

**Affiliations:** 1 Laboratory of Endocrinology, Department of Clinical Chemistry, Amsterdam Neuroscience, Amsterdam Gastroenterology, Endocrinology and Metabolism, Amsterdam University Medical Center, Location AMC, University of Amsterdam, Meibergdreef 9, Amsterdam, Netherlands; 2 Department of Endocrinology and Metabolism, Neuroscience Amsterdam, Amsterdam Gastroenterology, Endocrinology and Metabolism, Amsterdam University Medical Center, Location AMC, University of Amsterdam, Meibergdreef 9, K2-283, 1105 AZ Amsterdam, the Netherlands; 3 Metabolism and Reward Group, Netherlands Institute for Neuroscience, an Institute of the Royal Netherlands Academy of Arts and Sciences, Meibergdreef 47, Amsterdam, Netherlands

**Keywords:** Opioids, Blood glucose, Hyperglycaemia, Obesity, Diabetes mellitus, β-endorphin

## Abstract

**Purpose of Review:**

We are currently in the midst of a global opioid epidemic. Opioids affect many physiological processes, but one side effect that is not often taken into consideration is the opioid-induced alteration in blood glucose levels.

**Recent Findings:**

This review shows that the vast majority of studies report that opioid stimulation increases blood glucose levels. In addition, plasma levels of the endogenous opioid β-endorphin rise in response to low blood glucose. In contrast, in hyperglycaemic baseline conditions such as in patients with type 2 diabetes mellitus (T2DM), opioid stimulation lowers blood glucose levels. Furthermore, obesity itself alters sensitivity to opioids, changes opioid receptor expression and increases plasma β-endorphin levels.

**Summary:**

Thus, opioid stimulation can have various side effects on glycaemia that should be taken into consideration upon prescribing opioid-based medication, and more research is needed to unravel the interaction between obesity, glycaemia and opioid use.

## Introduction

We are currently in the midst of a global opioid epidemic, with numbers of opioid prescriptions continuously rising around the world [1]. Just in the USA alone, 153 million opioids were prescribed in 2019 [2], and many European countries, as well as Canada and Australia, also show yearly increasing numbers of opioid prescriptions [3]. Opioid-based analgesics were originally developed for short-term use as pain relief after surgery, or for palliative care and pain relief in late-stage cancer patients [4]. However, in the 1980s the (incorrect) idea that opioids were not as addictive as originally thought began to gain traction [4]. This led to a large increase in prescription for chronic non-cancer pain treatment, which is one of the main contributors to the current opioid epidemic [1, 5]. For example, in 2014 approximately 30 million adults in the USA received opioid-based medication for a non-cancer pain-related condition [6]. With so many individuals receiving opioid prescriptions, it is essential that we improve our understanding on the effect of long-term opioid use on overall physiology.

Opioids effects go far beyond pain relief, including effects on glucose metabolism. The first reports of morphine affecting glycaemia date back to 1922 [7]. Moreover, opioid abusers show reduced glucose tolerance [8–11], further underlining the relationship between opioids and glucose metabolism. This relationship is particularly relevant, as individuals with obesity, which are at greater risk of developing insulin resistance and T2DM, suffer more frequently from pain conditions and are therefore more likely to receive opioid-based medications [12]. Indeed, up to 28% of all opioid prescriptions in the USA is to individuals with overweight/obesity [13] and reducing obesity rates could lower the number of opioid users in the USA with 1.5 million [14]. Thus, it is crucial to increase our knowledge on the effects opioids have on glucose metabolism. This review will summarize the studies that have investigated the relationship between opioids and blood glucose levels, and will highlight the knowledge gaps that need to be addressed in the future.

## The Opioid System

The body produces 4 types of endogenous opioids: β-endorphin, leu- and met-enkephalin, dynorphin and the most recently discovered endomorphin [15–18]. These peptides are produced in the central and peripheral nervous system, in immune cells, the gastrointestinal system (both in the enteric nervous system, as well as in mucosal endocrine cells of the gut [19]) and in the vasculature. When produced in the anterior pituitary gland [20], or in peripheral immune cells [21], endogenous opioids can also be released into the circulation. Because of this, they function both as neurotransmitters (when released in the central or peripheral nervous system) and as hormones (when released into the blood stream). Opioid peptides bind with different affinities to one of three classical opioid receptors: the δ-opioid receptor, κ-opioid receptor and µ-opioid receptor [22, 23]. Like the expression of endogenous opioids themselves, opioid receptors can be found throughout the body, including the nervous system, in peripheral organs such as the liver or adrenal glands and on immune cells [24].

The term opioids encompasses all peptides that mimic the actions of endogenous opioids by binding to one of the opioid receptors. Opioids can be divided into naturally derived opiates such as morphine, semi-synthetic derivatives such as heroin and synthetic opioids such as methadone or fentanyl [25]. While morphine was the first opiate used for analgesia, being extracted first in 1806 [25], synthetic opioids are the main factor driving the current opioid epidemic [26]. In the USA, hydrocodone is the most prescribed opioid, followed by methadone, oxycodone and fentanyl [3]. The vast majority of these prescribed opioid analgesics bind with highest affinity to the µ-opioid receptor [27].

Endogenous opioids are involved in multiple physiological processes, but their role in pain processing has been studied most extensively. However, opioids in the central nervous system are also implicated with the neural control of emotion, reward and stress responses [28]. Furthermore, endogenous opioids can influence respiration, the gastrointestinal tract and the cardiovascular system [29]. In line with the widespread actions of endogenous opioids, opioid medication causes many side effects including respiratory depression, nausea, constipation and bradycardia [25]. Lastly, the effects of opioid receptor stimulation on glycaemia have been investigated, but the exact mechanisms and consequences for patients receiving opioid-based medication have not been outlined yet.

## Opioids Effects on Glycaemia

Most studies show that intravenous synthetic or endogenous opioids cause an increase in blood glucose [7, 30–47]. These hyperglycaemic effects occurred upon administration of morphine [30–35, 48], fentanyl [36, 37], the µ-opioid receptor-specific ligand [D-Ala2, N-MePhe4, Gly-ol]-enkephalin (DAMGO) [35, 38, 39] and the endogenous opioid β-endorphin [34, 40–46, 49, 50]. Observations were made in humans [41, 42, 50, 51], dogs [48, 49], cats [31, 33], sheep [35, 38], rabbits [47], rats [37, 40, 43] and mice [30, 32, 34, 39]. Lastly, most studies explored direct effects of opioid infusion on blood glucose, but hyperglycaemic effects of opioids were also observed during an insulin tolerance test [39] and glucose tolerance test [39, 44].

In order to comprehend the consequences for people receiving opioid prescriptions, it is important to assess the magnitude of changes in glycaemia. In four studies investigating the effect of intravenous infusion of β-endorphin in humans, responses ranged from an increase of 8.2 mg/dL (0.5 mmol/L) [51] to an increase of 37.8 mg/dL (2.1 mmol/L) [50]. More importantly, these increases were rather long lasting: a 30 s intravenous bolus of β-endorphin resulted in elevated glycaemia lasting over one hour [41] up to two hours [51]. Considering that opioids are frequently used multiple times throughout the day [52], these hyperglycaemic side effects can have serious consequences for maintenance of normal blood glucose levels. The effects appear to be dose dependent: the studies with the lowest dose also reported the smallest increase in glycaemia [41, 42, 51], and the highest dose given also resulted in the most pronounced increase [50]. However, in a small comparison study between 3 different dosages (2 subjects were tested for each dose) no statistical differences in hyperglycaemia were reported [51]. Furthermore, all these experiments were performed using intravenous infusion of β-endorphin; thus, there is a need for studies investigating the effects of oral opioid prescriptions on blood glucose.

While the hyperglycaemic effects of intravenous opioids are robust and highly reproducible, several other experimental conditions led to other observations. Firstly, the site of opioid receptor stimulation can affect the glycaemic response. Intravenous or intracerebroventricular infusion of opioids increased glycaemia [7, 30–46], but injection into the spinal canal lowered blood glucose in three studies in rats and mice [32, 53, 54], and intraperitoneal injection in mice had no effect on glycaemia [55], indicating that specific sites of stimulation can cause differential effects on glucose levels. Importantly, the dose–response curve might differ between routes as a dose of β-endorphin that had no effect when administered intravenously increased glycaemia when infused intracerebroventricularly in dogs [49]. Furthermore, at very low doses (injected intravenously in sheep), morphine can have hypoglycaemic effects [35, 38]. Lastly, during a somatostatin clamp in dogs where insulin and glucagon were artificially clamped at a constant level, β-endorphin lowered glucose production in the liver and morphine lowered blood glucose levels [48, 56], suggesting that the hyperglycaemic effects of opioid stimulation require fluctuations in pancreatic hormone release.

In addition, also blood glucose levels at the onset are important for the subsequent effects of opioid administration. For instance, baseline hyperglycaemia, as often found in patients with T2DM, drastically alters the effects opioids have on blood glucose levels. In obese rats and mice (that show glycaemic disturbances), both a single β-endorphin infusion [30, 57] and repeated infusions of a peripheral µ-opioid receptor agonist over the course of three days [58] lowered blood glucose levels (without altering food intake). These hypoglycaemic effects of opioids were confirmed in patients with T2DM [59, 60]. Two strong findings indicate that it is hyperglycaemia per se, and not obesity per se, that alters opioids’ effects on blood glucose concentrations. Firstly, in a Type 1 DM model where mice are not obese but do have severe hyperglycaemia, similar glucose-lowering effects of opioid stimulation were found [61–63]. Secondly, in subjects with obesity that were not hyperglycaemic, β-endorphin infusion increased glycaemia, similar to subjects with a normal weight [64]. On the contrary, in both subjects with diabetes and a normal bodyweight, the hyperglycaemia at baseline caused reductions in blood glucose upon β-endorphin infusion [65]. Lastly, in healthy subjects that were kept under hyperglycaemic conditions, β-endorphin infusion also lowered glycaemia [41]. Thus, when blood glucose levels are elevated at the onset of opioid stimulation, opioids have glucose-lowering properties.

Obesity itself, in the absence of hyperglycaemia, appears to have a twofold effect on opioid-induced changes in glycaemia, depending on the dose of opioid administered. At very low concentrations, β-endorphin increases blood glucose in subjects with obesity while having no effects on blood glucose levels in individuals with a healthy bodyweight [64]. A similar rise in glucose in subjects with a healthy bodyweight and in subjects with obesity was observed with moderate concentrations [66], whereas in the highest tested concentrations subjects with obesity showed a smaller increase in glycaemia than subjects with a healthy bodyweight [65]. These findings show that in normoglycaemic individuals with obesity, sensitivity to opioid stimulation is altered. Whether only the administered dose of opioids further modulates the response to opioids, or also the presence of other obesity-related comorbidities such as sleep apnoea, which can affect insulin sensitivity [67], is not yet known. More detailed research is needed to explain these findings and anticipation of these dose-dependent side effects of prescribed opioids on glucose metabolism in this population is recommended.

## Opioid Stimulation of Glucoregulatory Hormones

**Fig. 1:**
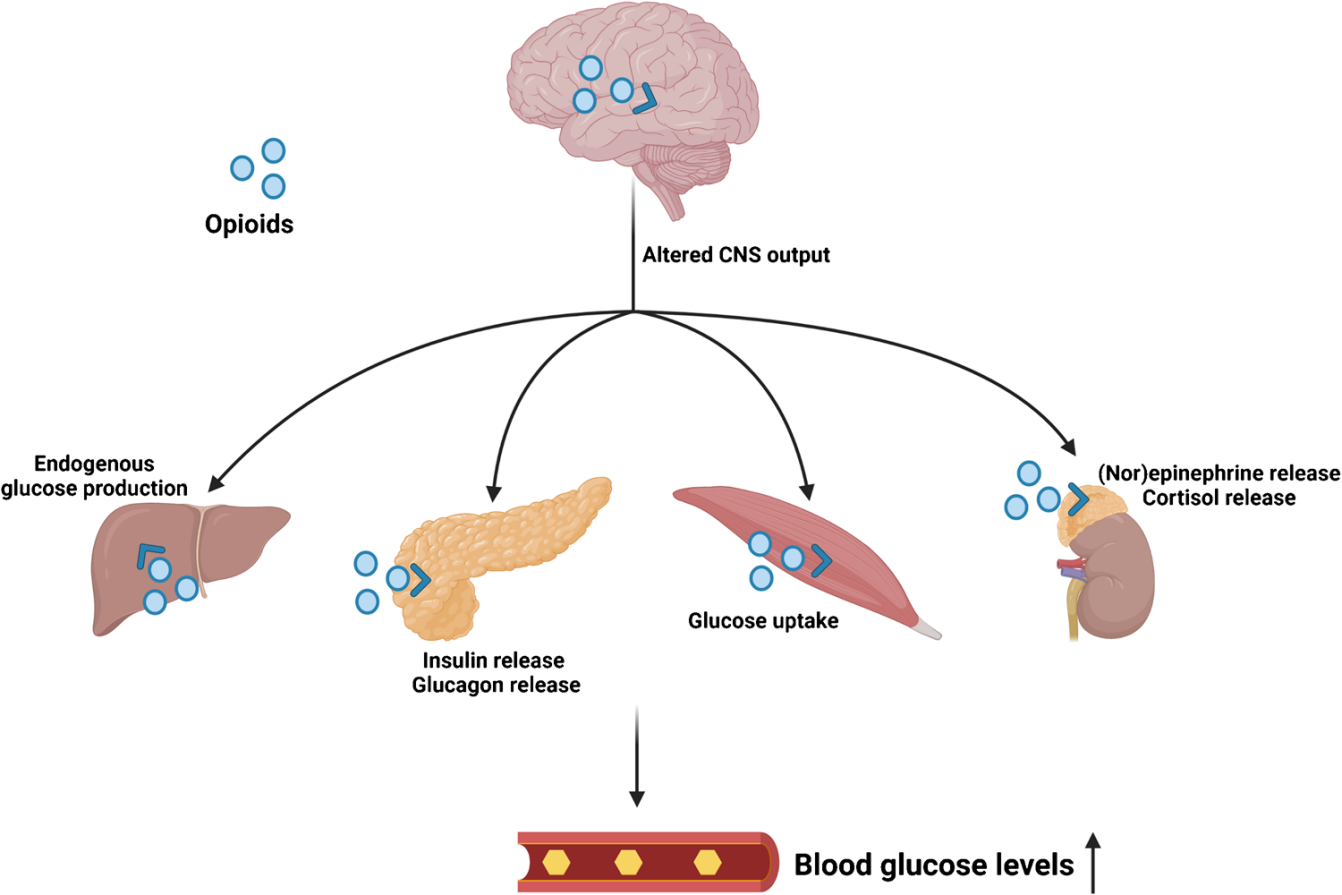
Physiological effects of opioid stimulation, resulting in hyperglycaemia. CNS = central nervous system

Opioid stimulation can increase glycaemic levels through a number of different pathways, including modulation of glucoregulatory hormones such as insulin, glucagon, epinephrine and cortisol (Fig. 1). Below we list what has been reported for opioid-induced changes in the release of these glucoregulatory hormones.

### Pancreatic Hormones

Opioids can modulate the release of pancreatic hormones by directly acting on opioid receptors in the pancreas, where particularly the µ- and δ-opioid receptor are expressed [68]. Furthermore, enkephalin [69] and β-endorphin are found within pancreatic islets [70, 71], suggesting that endogenous opioids may also be released locally to affect pancreatic hormone release. Lastly, opioids can alter pancreatic hormone release through central effects on the brain that cause alterations in sympathetic nervous system activity within the pancreas, as was shown for intracerebroventricular infusion of a µ-opioid receptor agonist [39].

For insulin, highly conflicting results have been found. A number of studies indicated that insulin secretion is reduced upon opioid infusion, thereby possibly mediating the hyperglycaemic effects that are usually observed [30, 39, 40, 43, 44, 46]. On the contrary, other studies found an increase in insulin release, which is attributed to an increase in blood glucose observed after opioid stimulation [37, 41, 42, 45, 48, 49, 55]. Studies on the direct effect of opioid receptor stimulation onto pancreatic islet isolates reported both an increase [72–76] and a decrease in insulin secretion [72, 73, 76, 77]. Several factors could explain the discrepancies found between these studies. Firstly, the extracellular glucose concentration can affect the insulin response [41, 55] and may have differed between studies. Secondly, depending on the concentration of the opioid given, different effects may be observed [42, 72, 73]. For example, low concentrations of an enkephalin analogue stimulated insulin release, whereas high concentrations inhibited insulin release at similar glucose concentrations [73]. Lastly, the type of opioid appears to have differential effects: in vitro application of enkephalin onto pancreatic islet cultures inhibited insulin release, whereas morphine administration stimulated it [76]. However, even when separating the in vivo studies for type of opioid given, still both increases and decreases in insulin release are reported. Future studies will have to unravel these discrepancies to better understand the regulatory effects of opioids on insulin secretion.

Glucagon was not measured in all studies, but the vast majority of studies that did measure glucagon indicate an increased secretion [40, 42–45, 48]. Only two studies found that glucagon did not change [37, 46]. Interestingly, in several studies, the increase in glucagon was seen in combination with enhanced insulin secretion [42, 45, 48]. This indicates that opioids can directly affect glucagon secretion, thereby overruling the suppressing effects of insulin on glucagon secretion. The effect of the opioid system on glucagon secretion might be responsible for the increase in blood glucose since glucagon stimulates hepatic glucose production.

Interestingly, the hypoglycaemic effects of opioids found in subjects that are hyperglycaemic at baseline provide more insights into the effects on pancreatic hormone release. Firstly, both in healthy subjects that were kept under hyperglycaemic conditions and Type II DM patients that suffer from high blood glucose levels, β-endorphin infusion stimulated insulin release, thereby lowering glycaemia [41, 59, 60]. This indicates that at least part of the hypoglycaemic effects of opioids can be attributed to opioids’ ability to stimulate insulin release. However, there are also insulin-independent effects on glycaemia that underlie the hypoglycaemic effects observed in the streptozotocin-induced Type I DM animal models [61–63]. For example, it appears that activation of the µ-opioid receptor increases glucose uptake by muscle cells [78], possibly through induction of protein kinase C-ζ activity that triggers glucose transporter 4 translocation to the cell membrane, thereby facilitating glucose uptake independent of insulin’s action [79, 80]. Interestingly, ob/ob mice (which are highly obese and hyperglycaemic) showed significantly more β-endorphin binding on muscle cells [81, 82]. If opioid receptor activation can stimulate glucose uptake through an insulin-independent pathway, greater opioid receptor binding in obese and hyperglycaemic mice could be an additional explanation for the hypoglycaemic effects in these mice. Whether the increased availability of opioid receptors indeed contributes to the glucose lowering effects, and whether this phenomenon is also present in human subjects with hyperglycaemia, remains to be determined. Furthermore, any opioid side-effects on glycaemia will likely occur through a combination of insulin-dependent and -independent effects.

One clear exception to the concept of both insulin-dependent and insulin-independent effects on glycaemia is the effects observed in Type 1 DM patients. Unfortunately, only one study to date has investigated whether opioids also have insulin-independent glucose-lowering effects in Type I DM patients. This study found that infusion of β-endorphin in insulin-using patients with DM highly stimulated glucagon release thereby increasing blood glucose levels [45]. However, while these patients were instructed to stop their medications 12 h before the experiment, the authors state that they likely still had exogenous insulin present in their circulation, as they had fasting glucose levels that were highly comparable to healthy individuals [45]. This complicates the comparison to the Type I DM animal models, because rats and mice used in these models have severe hyperglycaemia, and as previously outlined, glycaemic values before opioid infusion can dramatically alter opioids’ effects on blood glucose levels.

### Sympathetic Nervous System

Another pathway through which blood glucose levels can be increased is through activation of the sympathetic nervous system. Activation of the sympathetic nervous system causes release of the neurotransmitter norepinephrine from postganglionic neurons innervating various organs, and it also increases plasma levels of (nor)epinephrine that is produced in the medulla of the adrenal gland. Opioids may increase sympathetic nervous output through affecting the central control of the autonomous nervous system [83], at the level of the spine [84], or through direct release of (nor)epinephrine by the adrenal gland [85]. A highly consistent increase in plasma (nor)epinephrine levels upon opioid infusion is reported [36, 47–49, 83, 86–90].

Obesity itself is linked to increased sympathetic nervous system activation [91], possibly through the presence of comorbidities such as sleep apnea that can also enhance sympathetic tone [92]. Interestingly, obesity in the absence of hyperglycaemia affects the combined effects that β-endorphin and epinephrine have on glycaemia: the increase in glycaemia after combined infusion of β-endorphin and epinephrine was greater in individuals with obesity than in individuals with a healthy bodyweight, even though the glycaemic response to infusion of β-endorphin or epinephrine by itself did not differ between individuals with or without obesity [66]. Thus, it appears that obesity increases the synergistic effects of β-endorphin and epinephrine infusion on glycaemia. Whether the increased sympathetic tone during obesity also modulates the stimulating effects of opioids on sympathetic nervous system activity remains to be explored.

### Cortisol

Like the sympathetic nervous system, opioids can influence cortisol release through binding to receptors in the hypothalamus, but also through effects on the pituitary or the adrenal gland itself [93]. However, unlike the opioid effects on the sympathetic nervous system, opioid-induced changes in cortisol release are not consistent. Most importantly, it appears that there is a species difference in the effect opioids have on the release of glucocorticoids. For dogs and rats, a consistent increase in corticosterone release is found [36, 48, 49, 94–96], whereas in humans only a suppression of cortisol release is reported [97–100]. Therefore, cortisol is not likely to play a strong role in the consistently found hyperglycaemic effects of opioid stimulation in humans, as a decrease in cortisol is not expected to increase glycaemia. However, the decreased release of cortisol in response to opioid administration could play a role in the hypoglycaemic response to opioids that is seen when hyperglycaemia occurred at baseline. Whether the decrease in cortisol release upon opioid stimulation is the same in subjects that have hyperglycaemia, and whether reduced cortisol release indeed plays a factor in lowering glycaemia, remains to be investigated.

## Endogenous Opioid Release in Response to Changes in Glycaemia

While opioid administration affects glycaemia, changes in blood glucose levels also trigger a release of endogenous opioids, indicating a reciprocal relationship. For instance, hypoglycaemia, triggered by an insulin infusion, increases β-endorphin levels [101–106] and during exercise, when glucose is needed to fuel muscle activity, β-endorphin secretion into the blood is enhanced [107–110]. Interestingly, the rise in plasma β-endorphin levels during exercise is intensity and duration dependent, suggesting that metabolic needs influence the β-endorphin response [111]. Furthermore, β-endorphin also stimulates glucose uptake by the muscle and the sensitivity to β-endorphin is higher in contracted than non-contracted muscles [78]. Unlike hypoglycaemia, a rise in blood glucose levels (such as during an glucose tolerance test) did not increase plasma β-endorphin in most studies [112–116], or only mildly in some studies [117–119]. Thus, it appears that endogenous opioids are primarily released when there is an increased need for available glucose, such as during insulin-induced hypoglycaemia or during exercise, which is in line with the hyperglycaemic effects of opioid stimulation.

Interestingly, normoglycaemic subjects with obesity show increased baseline plasma β-endorphin levels [112–115, 118, 120, 121]. Furthermore, unlike in individuals with a healthy bodyweight, in subjects with obesity an increase in β-endorphin release was observed during a glucose tolerance test or after the consumption of a carbohydrate rich meal [112, 113, 117, 121, 122], though this effect was not reported in all studies [114, 117, 118]. Contradictory results have been found for the effects of DM on plasma β-endorphin levels. An increase in plasma β-endorphin concentrations specifically in non-insulin dependent patients was reported [123], whereas another study found no difference between non-insulin-dependent patients and controls [124]. Unfortunately, very little information about the type of DM or body weight is available for these two studies, thereby complicating the interpretation of these data. A third study investigating insulin-dependent patients with DM and a healthy body weight showed that both the normoglycaemic and the hyperglycaemic patients with DM had lower baseline β-endorphin concentrations and also found no increase in β-endorphin in response to exercise [125]. Thus, it is possible that in normal weight individuals DM lowers β-endorphin levels, whereas obesity in the absence of disruptions in blood glucose levels increases plasma β-endorphin concentrations.

As previously mentioned, obese mice had an increased number of µ- and δ-opioid receptors on skeletal muscle cells [81, 82]. Changes in opioid receptor expression in response to obesity in humans have only been reported in the brain, where a decrease in µ-opioid receptor availability in multiple brain areas was observed in individuals with morbid obesity [126, 127]. Whether these changes are the result of altered levels of endogenous opioids remains to be determined. Furthermore, a study in mice showed that binding of synthetic opioids to receptors on immune cells triggers the release of endogenous opioids [128], pointing to an interaction between synthetic and endogenous opioids. Thus, it would be of great interest to study how obesity-induced disruptions in the body’s endogenous opioid system affect the response to opioid medications.

## Future Directions

While many studies have explored the effects of opioid stimulation on glycaemia, a number of questions remain to be answered. Firstly, most studies investigated the effects of intravenous opioid administration. However, the majority of prescribed opioid medication is orally administered [25]. As we pointed out, administration route can affect the opioid-induced changes in glycaemia, and it is therefore crucial to further investigate the most common route of administration. Furthermore, the duration of analgesia for most prescribed opioids is much longer (around 4 h for most [25]) than in the studies that investigated the effect on blood glucose. Whether the effects on glycaemia also last several hours after oral opioid administration remains to be determined.

Secondly, the mechanisms by which opioid-induced hyperglycaemia occurs remain to be unraveled. Some studies have tried to narrow down how opioid stimulation increases blood glucose levels by investigating the direct effects on pancreatic hormone release, or by clamping pancreatic hormones to assess any direct effects of opioid stimulation. However, while isolating one element of opioids’ effects on the control of glycaemia provides detailed information about that element, it remains difficult to translate these findings back to a physiological setting. For example, during a somatostatin clamp, opioids lower endogenous glucose production in the liver [56], but concluding that therefore opioids will have glucose lowering effects is incorrect because the opioid-induced changes in for example glucagon release might outweigh the direct effects on endogenous glucose production. To truly unravel the mechanisms by which opioids affect glucose levels, more studies exploring different physiological situations (*e.g.* fasting or after a meal) are needed.

Thirdly, reviewing the current literature, we hypothesize that hyperglycaemia at the onset of opioid stimulation can alter the effects opioids have. This hypothesis will have to be further tested, including the mechanisms by which these changes occur. Furthermore, it appears that obesity can increase the sensitivity for opioid-induced hyperglycaemia, and it would be of interest to further pinpoint where in the development of insulin resistance during obesity this sensitivity for opioid-induced hyperglycaemia changes so that with higher levels of hyperglycaemia, opioids become hypoglycaemic. One step in this direction would be to further map the changes in central and peripheral opioid receptor expression, during obesity and DM.

## Conclusions and Implications

In conclusion, we described that opioids cause a significant, clinically relevant, long lasting increase in blood glucose. These findings were reproduced in many different studies, using different agonists, animal models and experimental settings. One important exception to the hyperglycaemic effects of opioids was found: when hyperglycaemia is present at baseline, opioids can actually lower glycaemia. The mechanisms by which these changes occur still require thorough investigation and studies should focus on opioid-induced alterations in hormone release as well as changes in insulin-dependent and insulin-independent glucose uptake. Glycaemia itself affects release of endogenous opioids, as during hypoglycaemia endogenous opioids are released into the circulation, indicating a reciprocal relationship. Lastly, obesity alters the sensitivity to opioids, opioid receptor expression and baseline endogenous opioid plasma concentrations. While many studies have explored how intravenous infusion of opioids affects glycaemia, substantial evidence on the effects of oral opioid-based medications on blood glucose levels is greatly needed. Nonetheless, based on the large number of studies confirming the hyperglycaemic effects of opioids, clinicians are encouraged to consider these possible side effects when prescribing opioid-based medications, particularly for patients with obesity and DM.

## References

[1] Degenhardt L (2019). Global patterns of opioid use and dependence: harms to populations, interventions, and future action. Lancet.

[2] Schieber LZ (2019). Trends and patterns of geographic variation in opioid prescribing practices by State, United States, 2006–2017. JAMA Netw Open.

[3] Board, I.N.C. Narcotic Drugs: Estimated World Requirements for 2019. 2018.

[4] DeWeerdt S (2019). Tracing the US opioid crisis to its roots. Nature.

[5] Boudreau D (2009). Trends in long-term opioid therapy for chronic non-cancer pain. Pharmacoepidemiol Drug Saf.

[6] Nahin RL (2019). Eighteen-year trends in the prevalence of, and health care use for, noncancer pain in the United States: Data from the medical expenditure panel survey. J Pain.

[7] Stewart GN, Rogoff JM (1922). Morphine, hyperglycemia and the adrenals. Am J Physiol Legacy Content.

[8] Reed JL, Ghodse AH (1973). Oral glucose tolerance and hormonal response in heroin-dependent males. Br Med J.

[9] Vescovi PP (1982). Glucose tolerance in opiate addicts. Diabetologia.

[10] Ceriello A (1987). Impaired glucose metabolism in heroin and methadone users. Horm Metab Res.

[11] Passariello N (1983). Glucose tolerance and hormonal responses in heroin addicts. A possible role for endogenous opiates in the pathogenesis of non-insulin-dependent diabetes. Metabolism.

[12] Okifuji A, Hare BD (2015). The association between chronic pain and obesity. J Pain Res.

[13] Stokes A (2020). Obesity and incident prescription opioid use in the U.S., 2000–2015. Am J Prev Med.

[14] Stokes A (2019). The contribution of obesity to prescription opioid use in the United States. Pain.

[15] Li CH, Chung D (1976). Isolation and structure of an untriakontapeptide with opiate activity from camel pituitary glands. Proc Natl Acad Sci U S A.

[16] Hughes J (1975). Identification of two related pentapeptides from the brain with potent opiate agonist activity. Nature.

[17] Goldstein A (1979). Dynorphin-(1–13), an extraordinarily potent opioid peptide. Proc Natl Acad Sci U S A.

[18] Zadina JE (1997). A potent and selective endogenous agonist for the mu-opiate receptor. Nature.

[19] Holzer P (2009). Opioid receptors in the gastrointestinal tract. Regul Pept.

[20] Young EA, Lewis J, Akil H (1986). The preferential release of beta-endorphin from the anterior pituitary lobe by corticotropin releasing factor (CRF). Peptides.

[21] Plein LM, Rittner HL (2018). Opioids and the immune system - friend or foe. Br J Pharmacol.

[22] Chang KJ (1979). Multiple opiate receptors: different regional distribution in the brain and differential binding of opiates and opioid peptides. Mol Pharmacol.

[23] Goldstein A, Naidu A (1989). Multiple opioid receptors: ligand selectivity profiles and binding site signatures. Mol Pharmacol.

[24] Wittert G, Hope P, Pyle D (1996). Tissue distribution of opioid receptor gene expression in the rat. Biochem Biophys Res Commun.

[25] Kerrigan S, Goldberger BA, Levine BS, Kerrigan S (2020). Opioids. Principles of Forensic Toxicology.

[26] Ayoo K (2020). The opioid crisis in North America: facts and future lessons for Europe. Anaesthesiol Intensive Ther.

[27] Madariaga-Mazon A (2017). Mu-Opioid receptor biased ligands: A safer and painless discovery of analgesics?. Drug Discov Today.

[28] Benarroch EE (2012). Endogenous opioid systems: current concepts and clinical correlations. Neurology.

[29] Shenoy SS, Lui F (2021). Biochemistry, endogenous opioids.

[30] Surwit RS (1989). Differential glycemic effects of morphine in diabetic and normal mice. Metabolism.

[31] Feldberg W, Shaligram SV (1972). The hyperglycaemic effect of morphine. Br J Pharmacol.

[32] Lux F, Brase DA, Dewey WL (1988). Differential effects of subcutaneous and intrathecal morphine administration on blood glucose in mice: comparison with intracerebroventricular administration. J Pharmacol Exp Ther.

[33] Borison HL (1962). Morphine-induced hyperglycemia in the cat. J Pharmacol Exp Ther.

[34] Park SH (2010). Characterization of blood glucose level regulation in mouse opioid withdrawal models. Neurosci Lett.

[35] Szeto HH (1995). Lack of relationship between opioid-induced changes in fetal breathing and plasma glucose levels. Am J Physiol.

[36] Ambrisko TD, Hikasa Y, Sato K (2005). Influence of medetomidine on stress-related neurohormonal and metabolic effects caused by butorphanol, fentanyl, and ketamine administration in dogs. Am J Vet Res.

[37] Johansen O (1994). Increased plasma glucose levels after Hypnorm anaesthesia, but not after Pentobarbital anaesthesia in rats. Lab Anim.

[38] Szeto HH (1995). Opioid modulation of fetal glucose homeostasis: role of receptor subtypes. J Pharmacol Exp Ther.

[39] Tuduri E (2016). Acute stimulation of brain mu opioid receptors inhibits glucose-stimulated insulin secretion via sympathetic innervation. Neuropharmacology.

[40] Fatouros IG (1997). Beta-endorphin infusion alters pancreatic hormone and glucose levels during exercise in rats. Eur J Appl Physiol Occup Physiol.

[41] Giugliano D (1988). Beta-endorphin-induced inhibition and stimulation of insulin secretion in normal humans is glucose dependent. Diabetes.

[42] Giugliano D (1987). Dual effect of beta-endorphin on insulin secretion in man. Horm Metab Res.

[43] Matsumura M (1984). In vivo and in vitro effects of beta-endorphin on glucose metabolism in the rat. Horm Metab Res.

[44] Giugliano D (1989). Beta-endorphin and islet hormone release in humans: evidence for interference with cAMP. Am J Physiol.

[45] Feldman M (1983). Beta-endorphin and the endocrine pancreas. Studies in healthy and diabetic human beings. N Engl J Med.

[46] Schleicher RL (1987). Beta-endorphin-induced hyperglycemia in rabbits: effects of a glucose or arginine challenge. Am J Physiol.

[47] May CN (1988). Intravenous morphine causes hypertension, hyperglycaemia and increases sympatho-adrenal outflow in conscious rabbits. Clin Sci (Lond).

[48] Radosevich PM (1984). Effects of morphine on glucose homeostasis in the conscious dog. J Clin Invest.

[49] Radosevich PM (1989). Central effects of beta-endorphins on glucose homeostasis in the conscious dog. Am J Physiol.

[50] Inder WJ (1996). The effect of beta-endorphin on basal and insulin-hypoglycaemia stimulated levels of hypothalamic-pituitary-adrenal axis hormones in normal human subjects. Clin Endocrinol (Oxf).

[51] Reid RL, Yen SS (1981). beta-Endorphin stimulates the secretion of insulin and glucagon in humans. J Clin Endocrinol Metab.

[52] Dowell D, Haegerich TM, Chou R (2016). CDC guideline for prescribing opioids for chronic pain - United States, 2016. MMWR Recomm Rep.

[53] Lux F (1989). Studies on the mechanism of hypoglycemia induced by intrathecal morphine: dissociation from behavioral effects, effects of tolerance and depletion of liver glycogen. J Pharmacol Exp Ther.

[54] Brase DA (1990). Hypoglycemia induced by intrathecal opioids in mice: stereospecificity, drug specificity and effect of fasting. J Pharmacol Exp Ther.

[55] Khawaja XZ, Green IC (1991). Dual action of beta-endorphin on insulin release in genetically obese and lean mice. Peptides.

[56] Radosevich PM (1984). Beta-endorphin inhibits glucose production in the conscious dog. J Clin Invest.

[57] Su CF (2004). Infusion of beta-endorphin improves insulin resistance in fructose-fed rats. Horm Metab Res.

[58] Tzeng TF (2007). Activation of mu-opioid receptors improves insulin sensitivity in obese Zucker rats. Life Sci.

[59] Giugliano D (1987). Beta-endorphin infusion restores acute insulin responses to glucose in type-2 diabetes mellitus. J Clin Endocrinol Metab.

[60] Giugliano D (1987). Beta-endorphin and islet hormone release in type-2 diabetes mellitus the effects of normoglycemia, enkephalin, naloxone and somatostatin. Diabete Metab.

[61] Cheng JT (2002). Plasma glucose-lowering effect of beta-endorphin in streptozotocin-induced diabetic rats. Horm Metab Res.

[62] Cheng JT (2001). Plasma glucose-lowering effect of tramadol in streptozotocin-induced diabetic rats. Diabetes.

[63] Liu IM (1999). Activation of opioid mu-receptor by loperamide to lower plasma glucose in streptozotocin-induced diabetic rats. Neurosci Lett.

[64] Giugliano D (1992). Physiological elevations of plasma beta-endorphin alter glucose metabolism in obese, but not normal-weight, subjects. Metabolism.

[65] Giugliano D (1987). Hyperglycemia and obesity as determinants of glucose, insulin, and glucagon responses to beta-endorphin in human diabetes mellitus. J Clin Endocrinol Metab.

[66] Giugliano D (1988). Altered metabolic and hormonal responses to epinephrine and beta-endorphin in human obesity. J Clin Endocrinol Metab.

[67] Yang D (2013). Effects of continuous positive airway pressure on glycemic control and insulin resistance in patients with obstructive sleep apnea: a meta-analysis. Sleep Breath.

[68] Peng J, Sarkar S, Chang SL (2012). Opioid receptor expression in human brain and peripheral tissues using absolute quantitative real-time RT-PCR. Drug Alcohol Depend.

[69] Polak JM (1977). Enkephalin-like immunoreactivity in the human gastrointestinal tract. Lancet.

[70] von Dorsche HH, Falt K, Zuhlke H (1989). Immunohistochemical investigations of beta-endorphin in human pancreatic islets. Acta Histochem.

[71] Zhang M, Zheng M, Schleicher RL (1992). Localization of beta-endorphin in rabbit pancreatic islets. Mol Cell Neurosci.

[72] Garcia-Barrado MJ (2002). Role of mu-opioid receptors in insulin release in the presence of inhibitory and excitatory secretagogues. Eur J Pharmacol.

[73] Green IC (1980). Effect of enkephalins and morphine on insulin secretion from isolated rat islets. Diabetologia.

[74] Curry DL, Bennett LL, Li CH (1987). Stimulation of insulin secretion by beta-endorphins (1–27 & 1–31). Life Sci.

[75] Paul A, Guven N, Dietis N (2014). Opioid receptor-dependent modulation of insulin-release in pancreatic beta-cells. UK J Pharm Biosci.

[76] Kanter RA, Ensinck JW, Fujimoto WY (1980). Disparate effects of enkephalin and morphine upon insulin and glucagon secretion by islet cell cultures. Diabetes.

[77] Schleicher RL (1989). Beta-endorphin inhibits insulin secretion from isolated pancreatic islets. Endocrinology.

[78] Evans AA, Khan S, Smith ME (1997). Evidence for a hormonal action of beta-endorphin to increase glucose uptake in resting and contracting skeletal muscle. J Endocrinol.

[79] Cheng KC (2013). Opioid mu-receptors as new target for insulin resistance. Pharmacol Ther.

[80] Yang TT (2009). Mediation of protein kinase C zeta in mu-opioid receptor activation for increase of glucose uptake into cultured myoblast C2C12 cells. Neurosci Lett.

[81] Hughes S, Smith ME, Bailey CJ (1992). Beta-endorphin and corticotropin immunoreactivity and specific binding in the neuromuscular system of obese-diabetic mice. Neuroscience.

[82] Evans AA, Smith ME (1996). Distribution of opioid peptide receptors in muscles of lean and obese-diabetic mice. Peptides.

[83] Gaumann DM (1988). Sympathetic stimulating effects of sufentanil in the cat are mediated centrally. Neurosci Lett.

[84] Romagnano MA, Hamill RW (1984). Spinal sympathetic pathway: an enkephalin ladder. Science.

[85] Castanas E (1985). Interaction of opiates with opioid binding sites in the bovine adrenal medulla: I. Interaction with delta and mu sites. J Neurochem.

[86] Hoehe M, Duka T (1993). Opiates increase plasma catecholamines in humans. Psychoneuroendocrinology.

[87] Kiritsy-Roy JA, Marson L, Van Loon GR (1989). Sympathoadrenal, cardiovascular and blood gas responses to highly selective mu and delta opioid peptides. J Pharmacol Exp Ther.

[88] Van Loon GR, Appel NM (1981). Plasma norepinephrine, epinephrine and dopamine responses to intracerebral administration of a met-enkephalin analog, D-ala2-met-enkephalinamide, in rats. Neuroendocrinology.

[89] Van Loon GR, Appel NM, Ho D (1981). beta-Endorphin-induced stimulation of central sympathetic outflow: beta-endorphin increases plasma concentrations of epinephrine, norepinephrine, and dopamine in rats. Endocrinology.

[90] Conway EL, Brown MJ, Dollery CT (1983). Plasma catecholamine and cardiovascular responses to morphine and D-ala2-d-leu5-enkephalin in conscious rats. Arch Int Pharmacodyn Ther.

[91] Thorp AA, Schlaich MP (2015). Relevance of sympathetic nervous system activation in obesity and metabolic syndrome. J Diabetes Res.

[92] Lombardi C, Pengo MF, Parati G (2019). Obstructive sleep apnea syndrome and autonomic dysfunction. Auton Neurosci.

[93] Pechnick RN (1993). Effects of opioids on the hypothalamo-pituitary-adrenal axis. Annu Rev Pharmacol Toxicol.

[94] Iyengar S, Kim HS, Wood PL (1987). Mu-, delta-, kappa- and epsilon-opioid receptor modulation of the hypothalamic-pituitary-adrenocortical (HPA) axis: subchronic tolerance studies of endogenous opioid peptides. Brain Res.

[95] Ignar DM, Kuhn CM (1990). Effects of specific mu and kappa opiate tolerance and abstinence on hypothalamo-pituitary-adrenal axis secretion in the rat. J Pharmacol Exp Ther.

[96] Gonzalvez ML, Milanes MV, Vargas ML (1991). Effects of acute and chronic administration of mu- and delta-opioid agonists on the hypothalamic-pituitary-adrenocortical (HPA) axis in the rat. Eur J Pharmacol.

[97] Zis AP (1984). Morphine inhibits cortisol and stimulates prolactin secretion in man. Psychoneuroendocrinology.

[98] McDonald R (1959). Effect of morphine and nalorphine on plasma hydrocortisone levels in man. J Pharmacol Exp Ther.

[99] Hoehe M, Duka T, Doenicke A (1988). Human studies on the mu opiate receptor agonist fentanyl: neuroendocrine and behavioral responses. Psychoneuroendocrinology.

[100] Pende A (1986). Evaluation of the effects induced by four opiate drugs, with different affinities to opioid receptor subtypes, on anterior pituitary LH, TSH, PRL and GH secretion and on cortisol secretion in normal men. Biomed Pharmacother.

[101] Kjaer A (1993). Insulin/hypoglycemia-induced adrenocorticotropin and beta-endorphin release: involvement of hypothalamic histaminergic neurons. Endocrinology.

[102] Petraglia F (1986). Effects of sodium valproate and diazepam on beta-endorphin, beta-lipotropin and cortisol secretion induced by hypoglycemic stress in humans. Neuroendocrinology.

[103] Nash JA (1989). Effects of naloxone on glucose homeostasis during insulin-induced hypoglycemia. Am J Physiol.

[104] Kinsley BT, Levy CJ, Simonson DC (1996). Prolactin and beta-endorphin responses to hypoglycemia are reduced in well-controlled insulin-dependent diabetes mellitus. Metabolism.

[105] Polinsky RJ (1987). Beta-endorphin, ACTH, and catecholamine responses in chronic autonomic failure. Ann Neurol.

[106] Mikines KJ (1985). The effect of training on responses of beta-endorphin and other pituitary hormones to insulin-induced hypoglycemia. Eur J Appl Physiol Occup Physiol.

[107] Radosevich PM (1989). Effects of low- and high-intensity exercise on plasma and cerebrospinal fluid levels of ir-beta-endorphin, ACTH, cortisol, norepinephrine and glucose in the conscious dog. Brain Res.

[108] Pierce EF (1993). Beta-endorphin response to endurance exercise: relationship to exercise dependence. Percept Mot Skills.

[109] Goldfarb AH (1990). Plasma beta-endorphin concentration: response to intensity and duration of exercise. Med Sci Sports Exerc.

[110] Goldfarb AH (1991). Beta-endorphin time course response to intensity of exercise: effect of training status. Int J Sports Med.

[111] Goldfarb AH, Jamurtas AZ (1997). Beta-endorphin response to exercise. An update. Sports Med.

[112] Scavo D (1987). Plasma beta-endorphin in response to oral glucose tolerance test in obese patients. Horm Metab Res.

[113] Giovannini C (1990). Beta-endorphin, insulin, ACTH and cortisol plasma levels during oral glucose tolerance test in obesity after weight loss. Horm Metab Res.

[114] Balon-Perin S (1991). The effects of glucose ingestion and fasting on plasma immunoreactive beta-endorphin, adrenocorticotropic hormone and cortisol in obese subjects. J Endocrinol Invest.

[115] Getto CJ (1986). Immunoreactive beta-endorphin increases after i.v. glucose in obese human subjects. Brain Res Bull.

[116] Farrell PA (1986). Plasma beta endorphin immunoreactivity: effects of sustained hyperglycemia with and without prior exercise. Life Sci.

[117] Getto CJ, Fullerton DT, Carlson IH (1984). Plasma immunoreactive beta-endorphin response to glucose ingestion in human obesity. Appetite.

[118] Stomati M (1998). Beta-endorphin response to oral glucose tolerance test in obese and non-obese pre- and postmenopausal women. Gynecol Endocrinol.

[119] Barreca T (1995). Plasma beta-endorphin levels and glucose tolerance in patients with chronic renal failure. Biomed Pharmacother.

[120] Margules DL (1978). beta-Endorphin is associated with overeating in genetically obese mice (ob/ob) and rats (fa/fa). Science.

[121] Zelissen PM (1991). Beta-endorphin and insulin/glucose responses to different meals in obesity. Horm Res.

[122] Brunani A (1996). Influence of insulin on beta-endorphin plasma levels in obese and normal weight subjects. Int J Obes Relat Metab Disord.

[123] Vermes I (1985). Increased plasma levels of immunoreactive beta-endorphin and corticotropin in non-insulin-dependent diabetes. Lancet.

[124] Burrin JM (1986). Plasma immunoreactive beta-endorphin in non-insulin-dependent diabetes. Lancet.

[125] Wanke T (1996). Defective endogenous opioid response to exercise in type I diabetic patients. Metabolism.

[126] Joutsa J (2018). Binge eating disorder and morbid obesity are associated with lowered mu-opioid receptor availability in the brain. Psychiatry Res Neuroimaging.

[127] Karlsson HK (2015). Obesity is associated with decreased mu-opioid but unaltered dopamine D2 receptor availability in the brain. J Neurosci.

[128] Celik MO (2016). Leukocyte opioid receptors mediate analgesia via Ca(2+)-regulated release of opioid peptides. Brain Behav Immun.

